# Ventricular Arrhythmia in Septal and Apical Hypertrophic Cardiomyopathy: The French-Canadian Experience

**DOI:** 10.3389/fcvm.2020.548564

**Published:** 2020-10-22

**Authors:** Christian Steinberg, Charles Nadeau-Routhier, Philippe André, François Philippon, Jean-François Sarrazin, Isabelle Nault, Gilles O'Hara, Louis Blier, Franck Molin, Benoit Plourde, Karine Roy, Eric Larose, Marie Arsenault, Jean Champagne

**Affiliations:** Division of Cardiology, Multidisciplinary Cardiovascular Department, Institut Universitaire de Cardiologie et Pneumologie de Québec (IUCPQ-UL), Université Laval, Québec City, QC, Canada

**Keywords:** hypertrophic cardiomyopathy, apical hypertrophic cardiomyopathy, ventricular arrhythmia, septal hypertrophic cardiomyopathy, French-Canadian

## Abstract

**Background:** Apical hypertrophic cardiomyopathy (aHCM) is thought to have a more benign clinical course compared to septal HCM (sHCM), but most data have been derived from Asian cohorts. Comparative data on clinical outcome in Caucasian aHCM cohorts are scarce, and the results are conflicting. The aim of this study was to estimate the prevalence and outcome of aHCM in French-Canadians of Caucasian descent.

**Methods and results:** We conducted a retrospective, single-center cohort study. The primary endpoint was a composite of documented sustained ventricular arrhythmia (VA), appropriate ICD therapy, arrhythmogenic syncope, cardiac arrest, or all-cause mortality. A total of 301 HCM patients (65% males) were enrolled including 80/301 (27%) with aHCM and 221/301 (73%) with sHCM. Maximal wall thickness was similar in both groups. Left ventricular apical aneurysm was significantly more common in aHCM (10 vs. 0.5%; *p* < 0.001). The proportion of patients with myocardial fibrosis ≥ 15% of the left ventricular mass was similar between aHCM and sHCM (21 vs. 24%; *p* = 0.68). Secondary prevention ICDs were more often implanted in aHCM patients (16 vs. 7%; *p* = 0.02). The primary endpoint occurred in 26% of aHCM and 10.4% of sHCM patients (*p* = 0.001) and was driven by an increased incidence of sustained VA (10 vs. 2.3%; *p* = 0.01). Multivariate analysis identified apical aneurysm and a phenotype of aHCM as independent predictors of the primary endpoint and the occurrence of sustained ventricular tachycardia. Unexplained syncope and a family history of sudden cardiac death were additional predictors for sustained VA. Apical HCM was associated with an increased risk of ventricular arrhythmia even when excluding patients with apical aneurysm.

**Conclusions:** The phenotype of apical HCM is much more common in French-Canadians (27%) of Caucasian descent compared to other Caucasian HCM populations. Apical HCM in French-Canadians is associated with an increased risk for ventricular arrhythmia.

## Introduction

Hypertrophic cardiomyopathy (HCM) is the most common hereditary cardiomyopathy with an estimated prevalence of 1:500 and is a leading cause of sudden unexplained death, in particular in young competitive athletes (1, 2). Depending on the predominant localization of segmental myocardial hypertrophy, different HCM phenotypes can be distinguished including septal HCM (sHCM) and apical HCM (aHCM) (3, 4). Apical HCM is characterized by focal hypertrophy of the apical segments resulting in a characteristic ace-of-spade-like end-systolic configuration of the left ventricle that is associated with deep, symmetric precordial T-wave inversions on the surface ECG (5). Additional findings may include midventricular obstruction/gradients and/or left ventricular apical aneurysm (6). The prevalence of aHCM varies among different ethnic groups and is usually more common in East-Asian and Afro-Caribbean populations compared to Caucasians (6–9). Apical HCM is considered to have a more benign course compared to sHCM, but most data are derived from Asian cohorts (10–12). There are conflicting data with regard to the outcome of aHCM in Caucasian North American populations (10, 13).

The French-Canadian population of the province of Quebec is predominantly of European Caucasian descent and represents a unique North American population from a genetic point of view. This feature is largely explained by the particular immigration history of Quebec together with regional colonization patterns resulting in founder mutations for various hereditary diseases (14).

The prevalence and outcome of aHCM in the Caucasian French-Canadian population is unknown, so far. The aim of this study was to estimate the prevalence of aHCM in the Caucasian French-Canadian population of Quebec and to assess the incidence and predictors of ventricular arrhythmia in this particular cohort.

## Methods

### Study Population and Definition of HCM

We conducted a retrospective cohort study at a single tertiary University center. The study was approved by the local ethics and review board. Eligible patients had to be ≥ 18 years old and were identified from the electronic medical records as well as the institutional cardiac imaging and pacemaker/defibrillator databases including the period from 2000 to 2017. To study our abovementioned hypothesis, we included only French-Canadians of Caucasian descent (self-reported ethnicity) in this study. Hypertrophic cardiomyopathy was diagnosed according to current guidelines and defined as otherwise unexplained left ventricular wall thickness ≥ 15 mm in one or more segments as measured by either transthoracic echocardiography or cardiac magnetic resonance imaging (CMR) (15–17). With regard to septal HCM, best efforts were made to include only patients with reverse curve septal hypertrophy. In the case of discordant imaging results with regard to HCM phenotype, maximal wall thickness, and presence/absence of apical aneurysm, we hierarchized CMR findings over echo results, which has also been proposed by recent literature (18). In the case of borderline segmental wall thickening (13–14 mm) HCM was diagnosed in the presence of at least one of the following findings: (1) positive genetic test for a known sarcomeric HCM variant, (2) a positive family history of HCM, (3) a ratio of end-diastolic septal/lateral wall thickness of ≥ 1.5, or (4) typical ECG findings of aHCM (16, 17). Apical HCM was defined as segmental hypertrophy of a ≥ 1 of the left ventricular apical segments with or without minor involvement of the inferoseptal or midseptal wall segments (13–14 mm). Dynamic obstruction of the left ventricular outflow tract, midventricular obstruction, and left ventricular apical aneurysm were defined according to current guidelines (4, 19). For the purpose of this study, we excluded mixed or diffuse forms of HCM, which represented <10% of the entire study cohort.

Patients were excluded in the presence of long-standing (≥ 5 years) uncontrolled arterial hypertension (systolic blood pressure ≥ 160 mmHg), moderate or severe aortic stenosis, subvalvular membranes of the left ventricular outflow tract, prosthetic aortic valve, evidence or strong suspicion of underlying HCM phenocopies including infiltrative cardiomyopathies (ex cardiac amyloidosis, Fabry disease, etc.), or left ventricular non-compaction.

### Cardiac Magnetic Resonance Imaging

Cardiac magnetic resonance imaging was performed using a 1.5-T system (Philips Achieva 1.5 Tesla, operating release 2.6 level 3, Philips Healthcare, Netherlands). All CMR exams were reviewed by a single experienced level-3 certified cardiologist who was blinded to the clinical data and myocardial phenotype of the patients. Myocardial fibrosis was quantified using validated software (CVI42, Circle, Canada) as previously described by our group (20). We set limits for myocardial fibrosis at ≥ 6 SD from the normal myocardium in accordance with published criteria for the assessment of myocardial fibrosis in hypertrophic cardiomyopathy (21, 22).

### Implantable Cardioverter Defibrillator (ICD) Implantation

The indications for ICD implantation were independently reevaluated by two independent electrophysiologists and assigned to primary or secondary prevention at the moment of the most recent follow-up visit according to current guidelines including traditional risk factors and new risk prediction models (16, 17). ICD programming including antitachycardia pacing (ATP) was standardized for all primary and secondary prevention ICDs with focus on delayed arrhythmia detection at higher ventricular rates based on available evidence and current guidelines (23–27). Supplementary Table 1 displays the ICD programming for primary prevention. Secondary prevention ICDs with documented ventricular fibrillation or polymorphic VT as index arrhythmia are programmed like primary prevention ICDs as recommended by current guidelines (26, 27). In the case of monomorphic VT with known rate, the slowest therapy zone is programmed 10 bpm below the documented tachycardia rate as recommended (26, 27).

### Arrhythmic Outcomes and All-Cause Mortality

The primary endpoint was a composite of documented sustained ventricular arrhythmia (VT) (monomorphic VT, polymorphic VT, or ventricular fibrillation) as index arrhythmia or during follow-up, any appropriate ICD therapy (ATP or shock), likely arrhythmogenic syncope, resuscitated cardiac arrest from ventricular arrhythmia, or all-cause mortality. Likely arrhythmogenic syncope was defined as sudden, otherwise unexplained loss of consciousness in the absence of dynamic obstruction of the left ventricular outflow tract, bradycardia, and absence of a neurovegetative prodrome/postdrome (28).

### ECG Analysis

Resting standard 12-lead ECGs (paper speed 25 mm/s, 10 mm/mV) showing intrinsic ventricular depolarization were eligible for analysis. We excluded ECGs with paced QRS complexes. Eligible ECGs were analyzed for the presence of symmetric precordial T-wave inversion, signs of early repolarization, and ECG criteria suggesting left ventricular apical aneurysm (convex ST elevation of ≥ 1 mm in ≥ 2 contiguous leads through V1–V4 associated with loss of giant T-wave negativity) as published before (29–32). Early repolarization pattern was defined according to published recommendations requiring a J-point elevation of ≥ 1 mm in ≥ 2 contiguous inferior and/or lateral leads while excluding leads V1–V3 (33). Ventricular paced rhythms and QRS duration ≥ 110 ms were excluded for the analysis of the ER pattern. Symmetric T-wave inversion of precordial leads required at least 2 leads with a T-wave amplitude ≥ 1 mm. T-wave inversion with amplitudes ≥ 10 mm was classified as giant T-waves according to published criteria (5, 34).

### Genetic Testing

Genetic data were collected from the medical records and analyzed if available. For the purpose of this study, we only included genetic testing using contemporary next-generation sequencing (NGS). DNA sequencing targeted all coding exons, all exon–intron boundaries, and some potential mutation sites located outside the coding regions of the target genes. Deletion/duplication analysis was performed for the majority of samples. The following 19 target genes for HCM and HCM phenocopies were selected: ACTC1, ALPK3, CSRP3, FLNC, GAA, GLA, LAMP2, MYBPC3, MYH7, MYL2, MYL3, PRKAG2, RAF1, SOS1, TNNI3, TNNT2, TPM1, and TTR. Variant classification was conducted according to current guidelines (35).

### Statistical Analysis

Categorical variables were analyzed using Fisher's exact test or χ^2^ test where appropriate. All continuous variables were tested for normality and are displayed as mean ± standard deviation (SD) or median with interquartile range (IQR) according to data distribution. Continuous variables were analyzed using Student's *t*-test or Mann–Whitney *U*-test where appropriate. Being survival-free from composite endpoints was compared using Kaplan–Meier curves and log-rank tests. Univariate and multivariate analyses for clinical predictors of the composite endpoint as well as sustained monomorphic ventricular tachycardia were conducted using logistic regression and a Cox proportional hazards model. Statistically significant univariate predictors with *p* ≤ 0.05 were selected for multivariate analysis. Statistical significance was considered for *p* < 0.05. All statistical analyses were conducted using Stata 14.1 Software (StataCorp, College Station, Texas, USA).

## Results

### Study Population

A total of 301 individuals with HCM were included in the study. Mean age was 55 ± 17 years, 65% were males, and all patients were Caucasians. Apical HCM was diagnosed in 27% (80/301) and sHCM in 73% (221/301) individuals. Baseline characteristics of the study population are displayed in Table 1. There were no differences between both groups with regard to age, sex, and medical comorbidities. Familial HCM accounted for 20% of all cases, and a family history of sudden cardiac death was present in 21% of the study population showing no difference between aHCM and sHCM. Overall, 36% of all study subjects had undergone ICD implantation with similar proportions of primary prevention ICDs in both groups (74% primary prevention ICDs). However, there were significantly more secondary prevention ICD carriers in the group of aHCM (16% vs 7%; *p* = 0.02). The subgroup of sHCM included 60 individuals who had undergone septal reduction therapy for previous, severe left ventricular outflow tract obstruction (Table 1).

**Table 1:** Baseline characteristics.

**∧**	**Total *N* = 301**	**Apical HCM *N* = 80**	**Septal HCM *N* = 221**	***P*-value**
Male, n (%)	197 (65)	57 (71)	140 (63)	0.22
Age at diagnosis, years	55 ± 17	57 ± 17	54 ± 17	0.39
BMI, kg/m2	28 ± 5	27 ± 4	29 ± 5	0.02
Familial HCM, n (%)	59 (20)	17 (21)	42 (19)	0.74
Family history of SCD, n (%)	62 (21)	20 (25)	42 (19)	0.26
Hypertension, n (%)	168 (56)	45 (56)	23 (56)	1.00
Dyslipidemia n (%)	152 (51)	42 (53)	110 (50)	0.70
Coronary artery disease, n (%)	54 (18)	12 (15)	42 (19)	0.50
Diabetes, n (%)	37 (12)	7 (9)	30 (14)	0.32
Active smoking, n (%)	21 (7)	8 (10)	13 (6)	0.21
Atrial fibrillation, n (%)	104 (35)	30 (38)	74 (33)	0.58
Atrial flutter, n (%)	40 (13)	13 (16)	27 (12)	0.44
CKD (≤60 mL/min], n (%)	28 (9)	9 (11)	19 (9)	0.50
**Septal reduction therapy**				
Any septal reduction, n (%)	60 (20)	0	60 (27)	<0.001
Septal myectomy, n (%)	45 (15)		45 (20)	
Septal ethanol ablation, n (%)	15 (5)		15 (7)	
**Cardiac implantable devices**				
ICD, n (%)	108 (36)	34 (43)	74 (33)	0.17
Primary prevention, n (%)	80 (27)	21 (27)	59 (27)	1.00
Secondary prevention, n (%)	28 (9)	13 (16)	15 (7)	0.02
Pacemaker, n (%)	44 (15)	9 (11)	34 (15)	0.46

*Continuous variables are presented as mean ± SD or absolute numbers and percentages where appropriate*.

*BMI, body mass index; SCD, sudden cardiac death; CKD, chronic kidney disease; ICD, implantable cardioverter defibrillator*.

### Findings of Cardiac Imaging

All individuals had at least one transthoracic echocardiogram, and in those with serial echocardiograms, the most recent exam in our database was used for analysis. The majority of patients with sHCM presented a reverse curve septal hypertrophy. In addition, 162/301 (54%) of all study subjects had at least one cardiac magnetic resonance (CMR) imaging during the study period. The proportion of patients with CMR was similar in both groups (aHCM 54 vs. sHCM 52%; *p* = 0.80). The results of cardiac imaging are outlined in Tables 2, 3. With regard to the maximal segmental wall thickness, there was no significant different between aHCM and sHCM. Indexed LV mass, biventricular volumes, and systolic function showed also no difference between both groups. The overall proportion of patients with detectable myocardial fibrosis was high (86%); however, scar volume and extension were similar in both groups. The median percentage of left ventricular fibrosis was 7% (IQR 10). The proportion of individuals with more advanced myocardial scarring (i.e., ≥ 15% of left ventricular mass) was slightly higher in patients with aHCM compared to sHCM, but the difference was not statistically significant. A left ventricular apical aneurysm was present in 10% of individuals with aHCM compared to only 0.5% of patients with sHCM (*p* < 0.001).

**Table 2:** Echocardiographic characteristics.

**Echocardiogram**	**All *N* = 301**	**Apical HCM *N* = 80**	**Septal HCM *N* = 221**	***P*-value**
Maximal wall thickness, mm	18 (5)	16 (5)	18 (5)	0.05
LVEF, %	60 (0)	60 (0)	60 0)	0.98
Presence of SAM, n (%)	221	3 (4)	118 (54)	<0.001
Any LVOT gradient, n (%)	151	9 (11)	142 (66)	<0.001

*Continuous variables are presented as median (interquartile range, IQR) according to distribution*.

*HCM, hypertrophic cardiomyopathy; LVEF, left ventricular ejection fraction; SAM, systolic anterior movement; LVOT, left ventricular outflow tract*.

**Table 3:** Cardiac magnetic resonance data.

**Cardiac MRI**	**All *N* = 158**	**Apical HCM *N* = 43**	**Septal HCM *N* = 115**	***P*-value**
Maximal wall thickness, mm	20 (6)	19 (6)	20 (6)	0.08
LV mass, g	175 (81)	176 (90)	174 (79)	0.64
LV mass indexed, g/m2	92 (35)	91 (36)	92 (35)	0.54
LVEDV mL/m2	73 (24)	73 (21)	73 (24)	0.83
LV ESV ml/m2	24 (19)	23 (19)	24 (19)	0.29
LVEF, %	75 (10)	75 (9)	76 (10)	0.22
RVEDV mL/m2	65 (24)	64 (27)	65 (23)	0.81
RVESV mL/m2	23 (11)	23 (17)	23 (10)	0.25
RVEF, %	64 (13)	64 (14)	64 (12)	0.35
Presence of any LGE, n (%)	136 (86)	39 (91)	97 (84)	0.44
Number of wall segments with LGE	4 (7)	5 (9)	4 (7)	0.21
LGE mass, g	12 (18)	9 (16)	13 (17)	0.52
LGE volume, mL	12 (18)	9 (18)	12 (18)	0.52
LGE percentage of LV, %	7 (10)	7 (9)	7 (10)	0.64
LGE volume ≥ 15 % LV), n (%)	30 (23)	9 (21)	28 (24)	0.68
LV apical aneurysm, n (%)	9 (3)	8 (10)	1 (0.5)	<0.001

*Continuous variables are presented as median (interquartile range, IQR)*.

*HCM, hypertrophic cardiomyopathy; LV, left ventricle/left ventricular; LVEDV, left ventricular end-diastolic volume; LVESV, left ventricular end-systolic volume; LVEF, left ventricular ejection fraction; RVEDV, right ventricular end-diastolic volume; RVESV, right ventricular end-systolic volume; RVEF, right ventricular ejection fraction; LGE, late gadolinium enhancement*.

### Arrhythmic Outcomes

**Figure 1:**
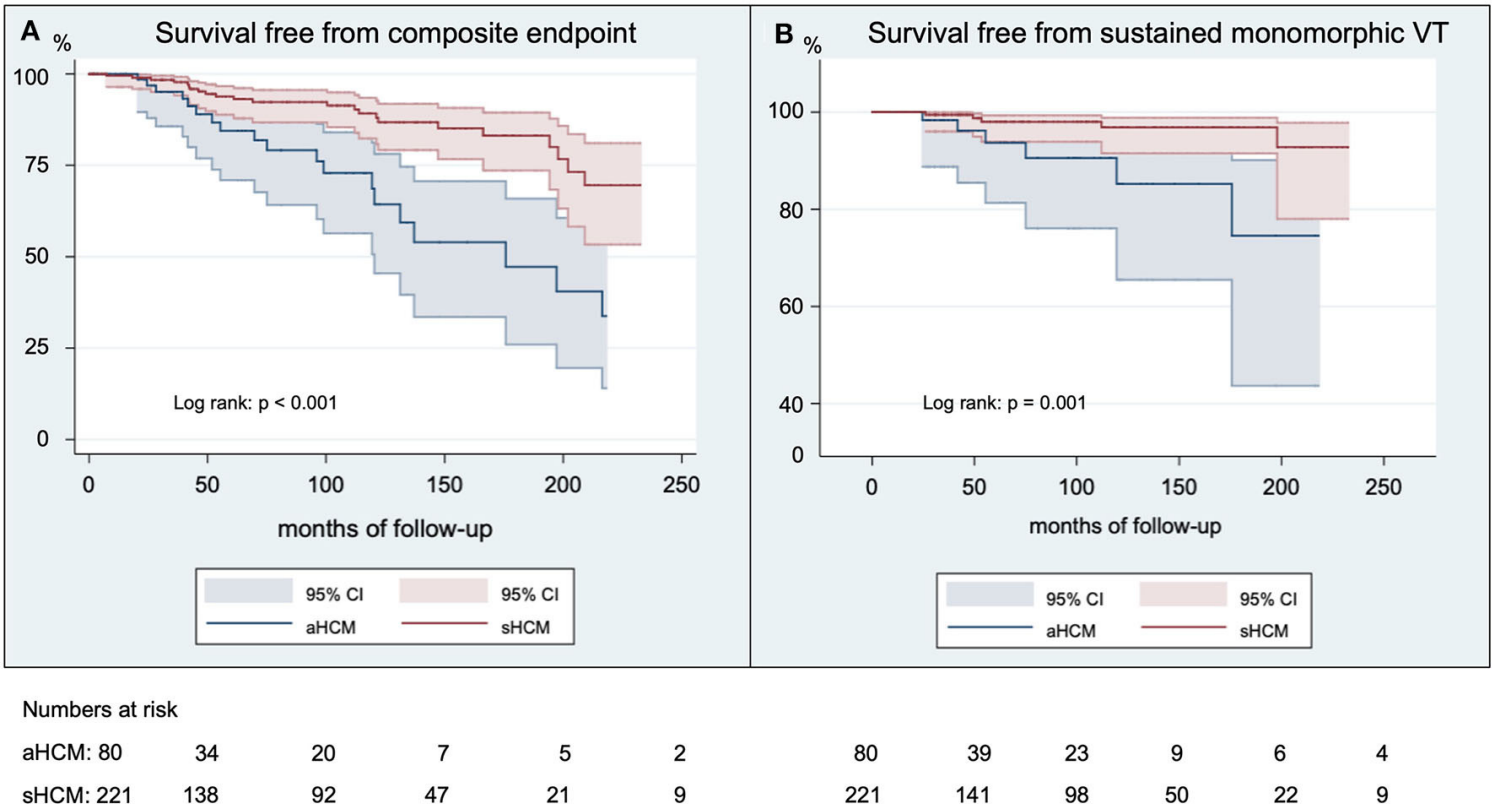
Survival free from arrhythmic outcomes. Shown are Kaplan-Meier curves for the survival free from the composite endpoint **(A)** and free from sustained monomorphic VT **(B)** in French Canadians with aHCM compared to sHCM. Kaplan-Meier curves are truncated at 250 months of follow-up.

**Figure 2:**
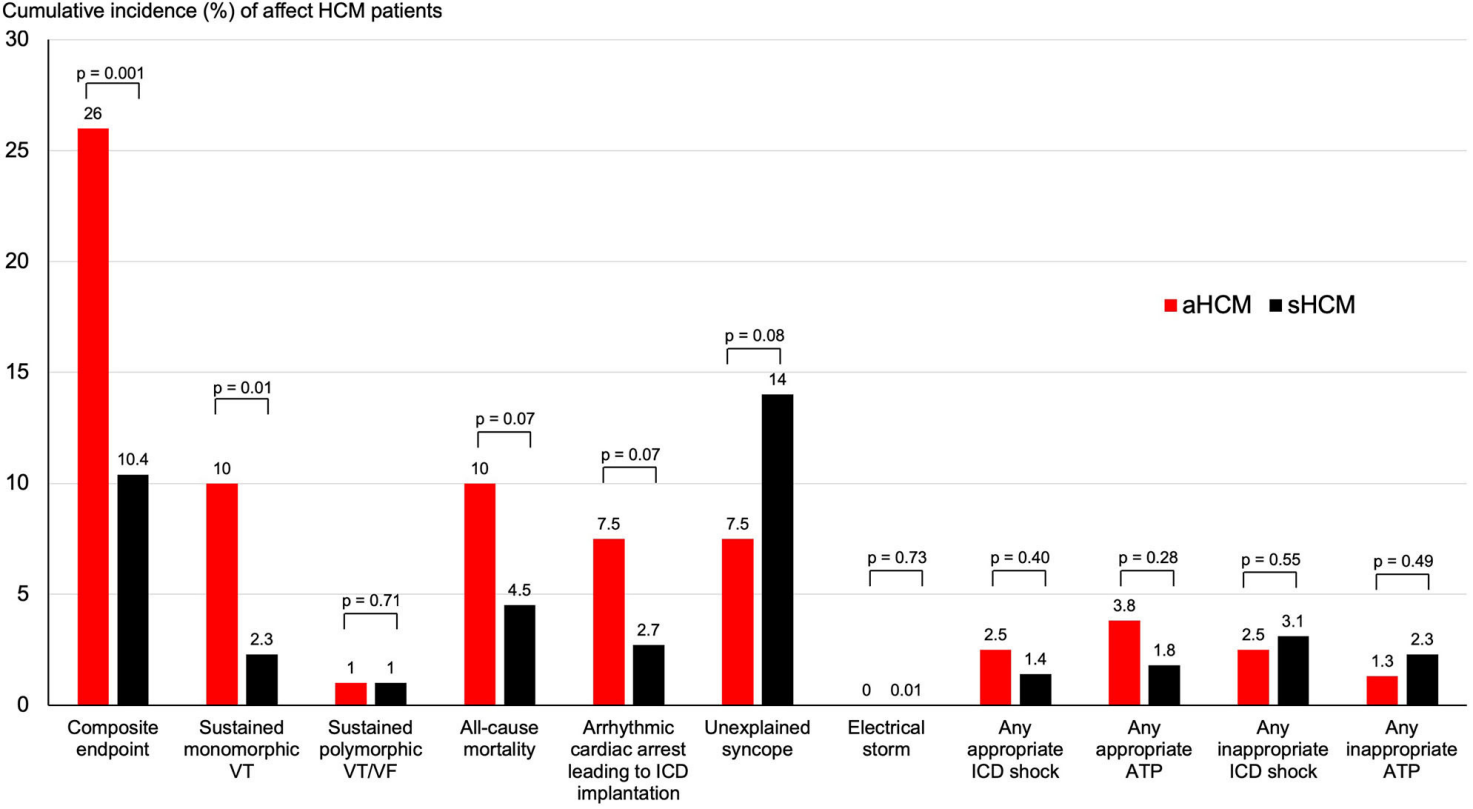
Cumulative incidence of arrhythmic outcomes and endpoints in apical and septal HCM. Shown are the cumulative incidence of mortality and arrhythmic endpoints over the follow-up period for patients with apical and septal HCM.

The median follow-up duration 47 months (IQR 87 months) for patients with aHCM and 77 months (IQR 111 months) for patients with sHCM. The follow-up duration was significantly longer for patients with sHCM (*p* = 0.02). Overall survival free from predefined endpoints is displayed in Figure 1A and was significantly decreased in patients with aHCM (log rank: *p* < 0.001) compared to sHCM. The median time to a first arrhythmic event of the composite endpoint was 48 months (IQR 109 months) for patients with aHCM and 56 months (IQR 80 months) for patients with sHCM (*p* = 0.83). A detailed distribution of arrhythmic events, all-cause mortality, and ICD therapies is illustrated in Figure 2. Arrhythmic events over time were significantly more common in individuals with aHCM. The combined primary endpoint occurred in 21/80 (26%) patients with aHCM compared to 23/221 (10.4%) with sHCM (*p* = 0.001). This was primarily driven by a significantly higher rate of sustained monomorphic VT occurring four times more often in aHCM compared to sHCM (10% vs. 2.3%; *p* = 0.01) (Figures 1B, 2). The 5-year incidence rate for the primary endpoint was 28.9% in aHCM and 11.9% in sHCM. The corresponding 5-year incidence of sustained monomorphic VT was 10.9% for patients with aHCM and 3.6% for patients with sHCM. Cardiac arrest as index event occurred in 7.5% of aHCM and 2.7% of sHCM patients (*p* = 0.07). Although the rate of index cardiac arrest was 2.8-fold higher in aHCM compared to sHCM, this difference did not reach statistical significance, which may be related to the overall small number of resuscitated cardiac arrests. Cardiac arrest as index event occurred at a similar median age in aHCM and sHCM (57 [14] vs. 56 [20] years; *p* = 0.89). Apical HCM was associated with significantly more arrhythmic events even when excluding cardiac arrest as index event (*p* = 0.01). With regard to the indication for initial ICD implantation, sustained monomorphic VT occurred in 19.4% of secondary prevention carriers compared to 8.8% of primary prevention carriers (*p* = 0.01). Patients with index cardiac arrest had a significantly increased risk of sustained monomorphic VT during follow-up (*p* = 0.04); however, 75% of all sustained VT events occurred in HCM individuals without initial cardiac arrest. Appropriate ICD therapies were similar in both groups, and the cumulative incidence of shocks and ICD was low. The overall rate of inappropriate ICD therapies (inappropriate shock or ATP) was low. A total of 9 inappropriate ICD shocks (3% per group, *p* = 1.0) and a total of 5 inappropriate ATP therapies (1% in aHCM and 2% in sHCM; *p* = 1.0) were recorded over the entire study period. Eighteen individuals died during the study period (6% overall mortality). The all-cause mortality rate in aHCM was more than twice as high compared to sHCM (1.5/100 patient-years vs. 0.6/100 patient-years), but this difference did not reach statistical significance. The vast majority out of hospital and without detailed information about the medical circumstances and a final ICD interrogation was not available for any of the deceased ICD carriers.

Those circumstances prevented any adjudication of the mortality causes. Cumulative all-cause mortality over time showed no difference between aHCM and sHCM (10% vs. 5%; *p* = 0.10), but the overall event rate was low. Interestingly, 20% (12/60) of sHCM patients post septal reduction therapy experienced a primary endpoint. Septal reduction therapy was not associated with an increased or decreased risk of an arrhythmic event or all-cause mortality on univariate analysis (HR 1.15 95% CI 0.56–2.35; *p* = 0.70).

### ECG Data

All eligible resting ECGs were analyzed to look for additional electrocardiographic markers that may correlate with the underlying HCM phenotype and the risk of arrhythmic events. ECG results are displayed in Table 4. PR intervals and QRS durations were slightly longer in individuals with sHCM. Conduction abnormalities were common and observed in 111/301 individuals (37%) including permanent ventricular pacing in 12% and complete left or right bundle branch block in 77/301 (26%) of all study subjects. Left bundle branch block was significantly more common in sHCM (19% vs. 1%; *p* < 0.001) and was typically related to previous septal reduction therapy (43 vs. 11%; *p* < 0.001). As expected, symmetric precordial T-wave inversion was significantly more common in aHCM compared to sHCM. The amplitude of T-wave inversion was also significantly more pronounced in aHCM. None of the HCM patients with confirmed left ventricular apical aneurysm displayed typical ECG findings, suggestive of aneurysm formation. Since a recent study has suggested an association between arrhythmic events and the co-presence of an early repolarization (ER) pattern, all eligible ECGs were in addition analyzed with regard to J-point abnormalities. An (ER) pattern was observed in 29/139 (21%) of all eligible ECGs with similar prevalence in both groups. The median J-point elevation was more pronounced in aHCM compared to sHCM (2 [2] vs. 1.5 [0.75] mm; *p* = 0.03). However, the presence of an ER pattern was not associated with an increased probability of ventricular arrhythmia in our study population (OR 1.19, 95% CI 0.46–3.07; *p* = 0.72).

**Table 4:** ECG data.

	**All *N* = 301**	**Apical HCM *N* = 80**	**Septal HCM *N* = 221**	***P*-value**
Heart rate, bpm	64 (12)	65 (12)	64 (13)	0.81
PR interval, ms	178 (46)	170 (40)	181 (49)	0.01
QRS duration, ms	100 (26)	98 (24)	104 (34)	0.02
QTc duration, ms	446 (43)	446 (43)	446 (43)	0.93
LBBB, n (%)	44 (15)	1 (1, 2)	43 (19, 4)	<0.001
RBBB, n (%)	30 (10)	4 (5)	26 (11, 8)	0.52
Ventricular paced QRS, n (%)	37 (12)	7 (8, 7)	30 (13, 5)	0.43
Precordial T-wave inversion V4–V6^$^, n (%)	103 (34%)	50(62,5)	52 (23, 5)	0.001
Maximal precordial T-wave inversion, mm	3 ± 4.5)	5.2 ±3,6	3.2 ± 2.3	0.39
**ER pattern in HCM**				
Eligible ECGs for analysis^¶^, n (%)	139 (46)	52 (65)	87 (39)	8,12
Presence of any ER pattern, n (%)^#^	29 (21)	10 (7)	19 (14)	0.41
Presence ERP inferior lead^#^	20 (14)	6 (4)	14 (10)	0,72
Maximal J-point elevation, mm	1.5 (1)	2 (2)	1 (0.75)	0.03

*Continuous variables are presented as mean ± SD or median (interquartile range, IQR) where appropriate*.

*QTc, corrected QT interval; LBBB, left bundle branch block; RBBB, right bundle branch block; ER, early repolarization; LV, left ventricular*.

¶ *To determine the presence of early repolarization pattern all ECGs with QRS ≥ 110 ms or paced QRS were excluded*.

# *Percentage of eligible ECGs for ER analysis*.

$ *Excluding ECGs with ventricular paced QRS, LBBB or LBBB-like non-specific intraventricular conduction delay*.

Interestingly, none of the individuals with confirmed left ventricular apical aneurysm had typical ER findings.

### Genetics

Although our study cohort includes patients from 2,000 on, routine genetic testing at our institution is only available since more recent. At the time of manuscript preparation, genetic data were available for 117 individuals (39%) with similar proportions of genetic testing in aHCM and sHCM (45% vs. 36%; *p* = 0.23) (Table 5). Genetic test results are displayed in Table 5. The overall yield of genetic testing was low, and a pathogenic or likely pathogenic variant was found in only 26 individuals (22%% of all tested individuals). Variants of MYBPC3 were most common accounting for 33% of all pathogenic/likely pathogenic mutations. The yield of genetic testing was significantly higher in sHCM compared to aHCM, and a pathogenic/likely pathogenic variant was found 5 times more often in sHCM (6% vs. 30%; *p* = 0.02). A positive family history of HCM did not influence the yield of genetic testing in this small genetic sample size (25% aHCM vs. 25% sHCM; *p* = 1.00), neither did the presence of a family history of SCD (6% aHCM vs. 6% sHCM; *p* = 1.00). The likelihood of a positive genetic test result remained unaffected by the absence or occurrence of arrhythmic events (*p* = 0.59).

**Table 5:** Targeted genetic testing using next-generation sequencing.

	**All HCM *N* = 301**	**aHCM *N* = 80**	**sHCM *N* = 221**	***P*-value**
Testing done, n (%)	117 (39)	36 (45)	81 (36)	0.23
Negative^#^, n (%) (of tested patients)	91 (78)	34 (94)	57 (70)	0.29
Tested patients with family history of HCM, n (%)	29 (25)	9 (25)	20 (25)	1.00
Tested patients without family history of HCM, n (%)	88 (75)	27 (75)	61 (75)	1.00
Tested patients with family history of SCD, n (%)	28 (24)	11 (31)	17 (21)	0.35
Pathogenic or likely pathogenic variants, n (%) (of tested patients)	26 (22)	2 (6)	24 (30)	0.02
Family history of HCM	13 (11)	9 (25)	20 (25)	1.00
No family history of HCM	13 (11)	27 (75)	61 (75)	
Family history of SCD	7 (6)	2 (6)	5 (6)	1.00

*HCM, hypertrophic cardiomyopathy; SCD, sudden cardiac death*.

# *Including benign/likely benign variances and variants of unknown significance (VUS) without evidence of pathogenicity after segregation studies*.

### Predictors of Arrhythmic Events

To identify risk factors of arrhythmic events, we conducted univariate and multivariate regression analysis using a Cox proportional hazard model. In addition to conventional risk factors (maximal wall thickness ≥ 30 mm, unexplained syncope, family history of SCD), we also included more recent risk factors (LGE, atrial fibrillation) and the presence and absence of left ventricular apical aneurysm. Given the low number of patients with segmental wall thickness ≥ 30 mm, we included grading of the maximal wall thickness as documented by CMR. The proportion of missing data about non-sustained ventricular tachycardia and/or exerciser-related drop of blood pressure prevented their inclusion for analysis. The results of the univariate analysis are displayed in Table 6. Apical HCM (HR 2.42, 95% CI 1.38–4.22; *p* = 0.002) and the presence of LV apical aneurysm (HR 3.70; 95% CI 1.12–12.27; *p* = 0.03) were predictors of the composite endpoint and the occurrence of sustained monomorphic VT. Even after exclusion of patients with left ventricular apical aneurysm, individuals with aHCM had significantly more arrhythmic events compared to sHCM (p = 0.004). A percentage of ≥ 15% of LGE was borderline predictive for the occurrence of a composite endpoint (HR 3.65 95% CI 0.98–13.62; *p* = 0.05). Among the conventional risk factors, unexplained syncope and a family history of unexplained SCD predicted sustained monomorphic VT at univariate analysis. Univariate predictors with a *p* ≤ 0.05 were entered into the multivariate analysis. Because of a strong collinearity between aHCM and the presence of a left ventricular apical aneurysm (*p* < 0.001), we conducted separate multiple regressions for those two factors. All other parameters did not show collinearity. Results of the multivariate analysis are displayed in Table 7. Apical HCM (HR 4.58, 95% CI 1.10–10.16; *p* = 0.04) and a percentage of left ventricular LGE ≥ 15% (HR 3.91, 95% CI 1.04–14.74; *p* = 0.04) were independent predictors of the composite endpoint in our study cohort. With regard to the occurrence of sustained monomorphic VT, aHCM (HR 5.17, 95% CI 1.65–16.15; *p* = 0.01), LV apical aneurysm (HR 4.89, 95% CI 1.20–19.81; *p* = 0.03), and a family history of unexplained SCD (HR 4.79, 95% CI 1.51–15.19; *p* = 0.01) were identified as independent predictors.

**Table 6:** Univariate analysis of predictors of arrhythmic outcomes.

**Factor**	**Hazard ratio**	**95% CI**	***P*-value**
**Composite endpoint**			
Apical HCM	2.42	1.38–4.22	0.002
LV apical aneurysm	3.70	1.12–12.27	0.03
Maximal segmental wall thickness (per mm)	0.93	0.82–1.05	0.25
Family history of unexplained SCD	1.07	0.44–2.59	0.88
Unexplained syncope	0.95	0.39–2.29	0.90
LGE ≥ 15%	3.82	1.21–12.08	0.02
Atrial fibrillation	1.38	0.71–2.71	0.35
Presence of ER pattern on ECG	0.95	0.29–3.12	0.94
**Sustained monomorphic VT**			
Apical HCM	5.89	1.78–16.90	0.003
LV apical aneurysm	6.20	1.64–23.40	0.01
Maximal segmental wall thickness	0.80	0.63–1.02	0.07
Family history of unexplained SCD	5.20	1.65–16.36	0.01
Unexplained syncope	3.34	1.05–10.66	0.04
LGE ≥ 15% of LV mass	7.41	0.77–71.27	0.08
Atrial fibrillation	0.74	0.23–2.34	0.61
Presence of ER pattern on ECG	1.85	0.40–8.44	0.43

*LGE, late gadolinium enhancement; ER, early repolarization; LV, left ventricular; SCD, sudden cardiac death*.

**Table 7:** Multivariate analysis of predictors of arrhythmic outcomes.

**Factor**	**Hazard ratio**	**95% CI**	***P*-value**
**Composite primary endpoint**			
Apical HCM	4.58	1.10–10.16	0.04
LV apical aneurysm	3.60	0.43–29.04	0.24
LGE ≥ 15% of LV mass	3.91	1.04–14.74	0.04
**Sustained monomorphic VT**			
Apical HCM	5.17	1.65–16.15	0.01
LV apical aneurysm	4.89	1.20–19.81	0.03
Unexplained syncope	2.63	0.76–9.07	0.13
Family history of unexplained SCD	4.79	1.51–15.19	0.01

*LV, left ventricular; LGE, late gadolinium enhancement; SCD, sudden cardiac death*.

## Discussion

Apical HCM is a distinct HCM phenotype with typical electrocardiographic and imaging findings that has been initially described in Japan where it accounts for 30–41% of all HCM cases (5, 6, 36). Over many years, aHCM was thought to affect predominantly Southeast Asian populations and to have a more benign clinical course compared to classic asymmetric sHCM (10, 36, 37). The findings of our study shed further light on the role of aHCM in Caucasian populations challenging the traditional concept of aHCM as a more benign phenotype.

First, we found that aHCM in the Quebec population was significantly more common than previously reported in other Caucasian populations accounting for 27% of all HCM cases followed at our center. Previous studies have reported prevalences of aHCM in the range of 3–11% among various Caucasian populations including the U.S. and the United Kingdom (7–9). This striking difference may be partially explained by differences in diagnostic criteria, infrequent use of cardiac magnetic resonance imaging in the past, or non-standardized classification of mixed/overlapping HCM phenotypes. For the present study, we excluded mixed HCM phenotypes and strictly applied the definitions of aHCM and sHCM as recommended by current guidelines (16, 17, 19). Additional reasons may be related to unique, yet unknown, genetic features of the French-Canadian population of Quebec (38, 39).

The most important observation of the present study is the significantly higher rate of ventricular arrhythmia in patients with aHCM compared to sHCM. The cumulative event rate in aHCM was 30%, which is among the highest rates reported so far for this particular HCM phenotype. The increased event rate in aHCM was largely related to a relatively high proportion of sustained monomorphic ventricular tachycardia (15%), which has not been reported before. Although aHCM was not associated with increased all-cause mortality compared to sHCM in our study, it caused increased morbidity due to the higher rate of recurrent ventricular arrhythmia.

Our results challenge the traditional concept of aHCM as a more “benign” form of HCM that has been previously reported in Asian and Non-Asian populations (10–12, 29). At least in the French-Canadian Caucasian population of Quebec, aHCM displays a more aggressive phenotype that is characterized by an increased risk of ventricular arrhythmia.

Previous studies reporting adverse cardiac events in patients with aHCM have mostly focused on all-cause mortality or included cohorts with different ethnic backgrounds (13, 40, 41). The study of Klarich et al. reported high all-cause mortality of 29% over a mean follow-up of 78 months that was mostly driven by non-cardiac deaths and the overall incidence of sudden cardiac death was low (13). The same study also identified female sex and atrial fibrillation as predictors of all-cause mortality, which was not reproduced by our study. Our study population was otherwise comparable to previous contemporary HCM cohorts with regard to age, medical comorbidities, and findings on cardiac imaging (8, 12, 13). The proportion of familial HCM and a positive family history of SCD was comparable to previous studies and was similar between aHCM and sHCM in our study (8). The high proportion of familial aHCM (20%) in our population is significantly higher than the 6% reported in a large Japanese cohort. This suggests different inheritance patterns and leaves room for speculation about the possibility of different pathophysiological and genetic mechanisms (42).

The increased risk of ventricular arrhythmia in our aHCM population also highlights the challenge of adequate risk stratification in aHCM. Preventive ICD implantation is currently recommended based on the presence of traditional risk factors or the more recent risk score developed by O'Mahony et al. (17, 28, 43). However, current risk prediction models were typically derived from patient populations with sHCM and may not be accurate for risk stratification in apical HCM.

The degree of segmental hypertrophy in aHCM and sHCM was similar in our study and did not show any differences with regard to the left ventricular mass index contrasting the results Kim et al. who found larger LV mass indices in sHCM (12). Those differences could be related to ethnic characteristics as the study of Kim et al. only included Korean individuals (12).

In addition to traditional risk factors, the presence of left ventricular myocardial fibrosis has been established as a prognostic factor for adverse cardiovascular outcomes in HCM with increased risk at scarring of ≥ 15 % of the left ventricular mass (22). In contrast to Asian HCM cohorts (12), the overall degree of left ventricular fibrosis in our study was similar between aHCM and sHCM. Interestingly, the proportion of aHCM patients with ≥ 15% of left ventricular fibrosis in our cohort was 10 times higher compared to the Korean aHCM cohort (12).

An important structural finding in our study cohort was a 10% prevalence of left ventricular apical aneurysm in the group of aHCM, which is among the highest reported in Caucasian cohorts with aHCM. Higher prevalences of apical aneurysm up to 23% have been described in Asian, but not Caucasian populations (12, 44). A crucial element for the assessment of left ventricular apical aneurysm is cardiac MRI, which has been shown to be more sensitive and accurate for the diagnosis of apical aneurysm compared to contrast-enhanced or standard echocardiography (45, 46). The lack of systematic CMR assessment of HCM patients in the past may have influenced the low prevalence overall and in aHCM in older studies. Even in our study, there are still 45% of study subjects who did not undergo CMR imaging—often because of previous implantation of a non-MRI compatible pacemaker or ICD. Therefore, the true proportion of LV apical aneurysm in our study cohort may actually be even higher. Apical aneurysm of the left ventricle has been identified as a predictor of worse prognosis and increased risk of ventricular arrhythmia in HCM (46, 47). However, most previous studies did not differentiate between aHCM and sHCM (46, 48). In our study, the presence of LV apical aneurysm was an independent predictor of ventricular arrhythmia, which is consistent with previous reports (46, 47). The presence of LV apical aneurysm is currently not part of recommended criteria for the indication of a primary prevention ICD (17, 43, 49). Our data and the reports of others, however, have shown that apical aneurysm is strongly associated with ventricular arrhythmia highlighting the need for a novel risk prediction model targeting specifically the subgroup of patients with aHCM.

## Limitations

Our study has several limitations. First, this is a retrospective single-center registry with limitations inherent to any retrospective study design.

Referral bias is a typical problem of registry data from tertiary centers and may have also influenced the present study. Because of that and a modest sample size, we cannot exclude that the true prevalence of aHCM may be different and that the overall cardiac risk in the general HCM population may be lower. It is possible that a subset of aHCM patients with very mild phenotypes and low cardiac risk never comes to medical attention. Second, the overall event rate in this study was relatively low, which may be related to the modest sample size, but is consistent with other large-scale contemporary studies and also reflects contemporary treatment strategies that have significantly improved the clinical course of HCM (50).

Inadvertent inclusion of hypertensive cardiomyopathies with isolated sigmoid hypertrophy of the basal septum is a potential concern for outcome analysis in all studies of sHCM cohorts and could potentially lower arrhythmic event rates. Despite very specific and detailed inclusion criteria, we cannot exclude that a small proportion of patients with sHCM actually may have hypertensive cardiomyopathy. True sHCM is typically characterized by reverse curve septal hypertrophy representing the majority of sHCM cases in our study. This phenotype should be distinguished from isolated sigmoid hypertrophy of the basal septum, which represents a more benign form and is often no true primary HCM (51).

A detailed mortality comparison between aHCM and sHCM was not possible given the low rate of all-cause mortality and the fact that deaths could not been adjudicated. Third, our detailed cardiac MRI analysis did not find significant differences between aHCM and sHCM in our French-Canadian population. Only 50% of our study population had a cardiac MRI performed, so it is possible that we may have missed differences in scar burden between the two study groups. Missing MRI studies were predominantly related to the presence of non-MRI compatible pacemaker or ICD systems and limited access to MRI until the early 2000's. Despite that, the proportion of patients with cardiac MRI was still higher than in other contemporary cohorts of aHCM patients (8, 13).

Only 39% of individuals underwent genetic testing, which mostly reflects missing genetic infrastructure at our center in the past. Although the genetic yield in aHCM is expected to be lower compared to sHCM, the results of our study were even lower compared to data from the literature (6). This may be partially explained by the overall low rates of genetic testing, but we cannot completely exclude the presence of an unidentified genetic substrate in the French-Canadian population.

## Conclusions

The phenotype of apical HCM is much more common in French-Canadians (27%) of Caucasian descent compared to other Caucasian HCM populations. Apical HCM in French-Canadians is associated with an increased risk for ventricular arrhythmia.

## Data Availability Statement

The authors acknowledge that the data presented in this study must be deposited and made publicly available in an acceptable repository, prior to publication. Frontiers cannot accept a article that does not adhere to our open data policies.

## Ethics Statement

The studies involving human participants were reviewed and approved by ERB of the IUCPQ-UL. Written informed consent for participation was not required for this study in accordance with the national legislation and the institutional requirements.

## Author Contributions

CS designed and planned the study. Data collection was performed by CS, CN-R, and PA. Data analysis and statistical analysis was performed by CS, CN-R, PA, and J-FS. Cardiac MRI interpretation was performed by EL. Interpretation of echocardiographies was performed by MA. CS wrote the manuscript that was subsequently revised and edited by all co-authors. All authors have read and approved the final version of the manuscript.

## Conflict of Interest

The authors declare that the research was conducted in the absence of any commercial or financial relationships that could be construed as a potential conflict of interest.

## Abbreviations

AF: Atrial Fibrillation
CMR: Cardiac magnetic resonance
ER: Early repolarization
aHCM: Apical hypertrophic cardiomyopathy
HCM: Hypertrophic cardiomyopathy
sHCM: Septal hypertrophic cardiomyopathy
LV: Left ventricle
LVEF: Left ventricular ejection fraction
ICD: Implantable cardioverter defibrillator
VF: Ventricular fibrillation
VT: Ventricular tachycardia
SCD: Sudden cardiac death.
