# Supplementary material for: Ventricular Arrhythmia in Septal and Apical Hypertrophic Cardiomyopathy: The French-Canadian Experience
Source: Front Cardiovasc Med. 2020 Oct 22;7:548564. doi: 10.3389/fcvm.2020.548564 (PMC7642600; doi:10.3389/fcvm.2020.548564)
Supplement: Supplementary file 1 [file Table_1.DOCX]

**Supplementary Table 1. Recommended ICD programming**

|  | **Monitor zone** | | **VT zone** | | **VF zone** | |
| --- | --- | --- | --- | --- | --- | --- |
|  | Rate (bpm) | Detection delay | Rate (bpm) | Detection delay | Rate (bpm) | Detection delay |
| **Boston Scientific** | 170-199 | 30 s | 200-249 | 10 s | ≥ 250 | 3 s |
| **Medtronic** | 170-199 | 36 intervals | 200-249 | 30/40 intervals | ≥ 250 | 30/40 intervals |
| **Abbott**  **(St-Jude Medical)** | 170-199 | 36 intervals | 200-249 | 30/40 intervals | ≥ 250 | 30/40 intervals |

Two ATP interventions in the VT zone were recommended.

| **ATP 1** | **Burst or Scan^&^** |
| --- | --- |
| Number of cycles | 4 |
| Pulses per burst |  |
| Initial | 10 |
| Increment | 1 |
| Maximum | 12 |
| Coupling interval | 84% |
| Decrement | 0 ms |
| Burst CL | 84% |
| Ramp decrement | 0 ms |
| Scan decrement | 20 ms |
| Minimum interval | 200 ms |

| **ATP 2** | **Burst or Scan** |
| --- | --- |
| Number of cycles | 4 |
| Pulses per burst |  |
| Initial | 10 |
| Increment | 1 |
| Maximum | 12 |
| Coupling interval | 81% |
| Decrement | 0 ms |
| Burst CL | 81% |
| Ramp decrement | 0 ms |
| Scan decrement | 20 ms |
| Minimum interval | 200 ms |

^&^Prefer Scan over Burst where programmable; in SJM: activate “readaptive”
